# Mapping loneliness through comparative analysis of USA and India using social intelligence analysis

**DOI:** 10.1186/s12889-023-17630-3

**Published:** 2024-01-22

**Authors:** Hurmat Ali Shah, Mowafa Househ

**Affiliations:** College of Science and Engineering, Hamad Bin Khalifa University, Ar-Rayan, Qatar

**Keywords:** Loneliness, Sentiment analysis, Mental health, Twitter

## Abstract

Loneliness, a widespread global public health concern, has far-reaching implications for mental and physical well-being, as well as economic productivity. It also increases the risk of life-threatening conditions. This study conducts a comparative analysis of loneliness in the USA and India using Twitter data, aiming to contribute to a global public health map on loneliness. Collecting 4.1 million tweets globally in October 2022 containing keywords like “lonely”, “loneliness”, and “alone”, the analysis focuses on sentiment and psychosocial linguistic features. Utilizing the Valence Aware Dictionary for Sentiment Reasoning (VADER) for sentiment analysis, the study explores variations in loneliness dynamics across cities, revealing geographical distinctions in correlated topics. The tweets with negative sentiment were further analyzed for psychosocial linguistic features to find a meaningful correlation between loneliness and socioeconomic and emotional themes and factors. Results give detailed top correlated topics with loneliness for each city. The results showed that the dynamics of loneliness through the topics correlated vary across geographical locations. Social media data can be used to capture the dynamics of loneliness which can vary from one place to another depending on the socioeconomic and cultural norms and sociopolitical policies. Social media data to understand loneliness can also provide useful information and insight for public health and policymaking.

## Introduction

Every year 1,62,000 Americans die from loneliness and social isolation (https://www.bbrfoundation.org/blog/epidemic-loneliness-and-despair-how-wisdom-can-help). Every forty seconds someone around the world commits suicide while loneliness is shown to be a direct cause of suicide [1]. In numbers that is equal to 8 million people dying per year. In England, 25 million people are lonely, and loneliness has been shown to increase the risk of death by 26% [2]. Loneliness not only affects the quality of life but also leads to other mental health issues thus burdening the public health service system. The monetary loss as result of loneliness is estimated to be between USD 8,074.80 and USD 12,077.70 per person per year in the United Kingdom [3]. The monetary cost of lost days and loss in productivity is estimated to be USD 3.14 billion per year for employees in the United Kingdom. Loneliness is shown to be associated with a high risk for multiple health conditions such as physical and mental health, dementia, and early mortality [4].

The causes of mental health issues can vary from genetic, social, and economic as well as immediate family or finding meaning in life. The result can be withdrawal from human bonds and touch. Loneliness can be helped through different interventions. But there is a need to understand the prevalence of loneliness to devise such technology-based and community-oriented strategies. Technology may have resulted in fragmented and individualized existence, but technology also can be a great healer. There are interventions to fight off loneliness that use technology from communication-based therapy to chatbots to robots to mobile apps. To heal loneliness and make people come out of their silent suffering through designing interventions that connect them to others.

In this paper, we carry out a comparative analysis of Twitter data on loneliness from the USA and India. This comparison is important because it will give us an insight into the varying and dynamic nature of loneliness in a developed country and a developing country. India and USA have the second and third largest populations in the world. China, the biggest country per population, does not give open access to Twitter as compared to India and the USA. Other social media apps such as Baidu chat are more popular in China. Comparative analysis of loneliness through Twitter data between USA and India also follows practice in the public health domain where multiple aspects of healthcare and health systems and policies of the two countries are compared. This paper studies how loneliness is experienced in both countries. This study is important because loneliness is closely related to mental health issues. Understanding the different expressions of loneliness and the possible causes in these two different cultural and social contexts will give insight into the dynamic and complex nature of loneliness backed by analysis through data.

This paper is part of the early stages of building the loneliness map. This paper can be seen as proof of the concept of the loneliness map and to drive home the point that a loneliness map is required as loneliness is felt differently across geographical variations. The data and analysis provided in this paper, although limited, can prove the concept that loneliness is associated with different socioeconomic and personal-emotional factors and themes across different geographical locations. The themes associated with loneliness can correspond to a range of socioeconomic and personal-emotional factors. This paper provides the starting point for further investigation into loneliness in the mentioned geographies based on thematic focus and associations.

Developing a detailed loneliness map that can reflect accurately the thematic causes of loneliness is a resource and time intensive project. When this project is taken especially on the basis of social media data, the available data can become intractable for analysis even for one country. It must be noticed here that this paper does not claim to provide scientific and empirical evidence such that it can be used directly. The paper presents direction for further research on loneliness in the mentioned geographies and the thematic associations. This paper uses Twitter data to compare the varying nature of loneliness across India and the USA. The remaining parts of the map, i.e., using multiple data sources and analyzing different regions and countries exhaustively will be carried out stepwise. Rather than using multiple sources of data we first focus on Twitter because the data it provides is diverse as well as from a limited dataset multiple insights can be gained as the users have to express themselves in limited characters. We collected data mentioning keywords associated with loneliness through Twitter API for researchers.

To develop the first part of the loneliness map, we use sentiment analysis of Twitter data through a natural language processing (NLP) tool. This is based on a psycho-linguistic model of understanding mental health issues. The collected tweets are stored in a database and then sentiment analysis using valence aware dictionary for sentiment reasoning (VADER) [5] tool from the natural language toolkit (NLTK) is carried out. VADER is a lexicon and rule-based model for sentiment analysis. The lexicon-based approach means that the algorithm is constructed using a dictionary that contains a detailed list of sentiment features. In addition, VADER also complements the lexicon-based dictionary with grammatical rules that are heuristic in nature. These rules complement the lexicon-based sentiment analysis to determine the polarity of the sentiment. The result of the sentiment analysis tool gives us an indication of loneliness in the dataset.

Summarily, the contributions of this paper can be given as;

1. Exploring social media data as a source to investigate how feelings of loneliness are expressed.
2. Investigating whether expressions of loneliness differ across geographic regions.
3. How the data on the expression of loneliness on social media can be divided into different categories and themes across the geographic divisions and investigating how these categories can vary geographically.

## Literature review

There have been several systematic and scoping reviews done to understand loneliness better. Understanding loneliness theoretically and its relation to mental health has been the result of several studies [6, 7]. The theoretical studies on loneliness have established the negative effects of loneliness. Similarly, from a health informatics perspective, there have been multiple systematic reviews of the connection between loneliness and mental health [8]. From the health informatics side, there also have been studies that deal with the application of technology-based intervention to cope with loneliness. There are also scoping reviews to find the effectiveness of technology-based intervention strategies for loneliness [9]. Loneliness is shown by these studies to be associated with an increased risk of mental health issues. Interventions for loneliness which are based either on technology or through building community were shown to be effective in reducing the negative effects of loneliness.

The social-psychological contexts of loneliness result in lower access to public healthcare. Technology is used to fill in the gap created by lack of access to a healthcare professional or service. There are scoping reviews done for information and communication technologies (ICT) based programs and interventions for older people. Byrne et al. carried out a scoping review of reviews to study the effectiveness of communication technologies to reduce the feeling of loneliness in older people [10]. The review included 28 studies, which combined covered 248 primary studies spanning over 50 years The paper concluded that interventions to counter loneliness based on communication technologies are effective. However, the study also found that there is lack of evidence and limited insight into innovative technologies like extended reality frameworks. Hards et al., studied through a systematic review and meta-analysis of digital technologies-based intervention to reduce loneliness in older adults [4]. The study analyzed 6 articles finally with 646 participants reported in combined. The study showed no statistical difference between the effectiveness of digital interventions, but it self-reported the lack of enough studies and the small sample size of participants to be the cause for the lack of validating effectiveness.

However, there are studies like [11] which establish the relationship between communication-based technologies and a reduction in the feeling of loneliness. Through a cross-sectional study of 4,315 older adults, aged above 50, it was reported that ruler older adults who used technology less frequently felt loneliness more than urban older adults. The study also explored the relationship between race, urbanity and age. The study recommended that technology-based intervention should consider social technology to address rural and racial disparities. Nevertheless, the connection or direct co-relation between social media technology use and a reduction in feelings of loneliness is not strongly established. Choi and Lee carried out a study of the effectiveness of social networking sites usage in older people for reducing loneliness [8]. The study analyzed 10 observational studies and five experimental studies, where five studies focused on loneliness and social isolation. Among the observational studies, some evidence was found that the use of social networking sites was associated with a reduction in feeling of loneliness and reduction in feeling of depression. However, the studies lacked on the experimental side.

The brief literature review provided above provides the scientific foundation for effectiveness of technology-based intervention in loneliness. However, there is a gap in global understanding and prevalence of loneliness. Surkalim et al., carried out a study of the prevalence of loneliness in 113 countries to identify data availability, gaps, and patterns for population level existence of loneliness [11]. However, the study neither designed a tool, nor an intervention based on the meta-analysis was carried out. To guide technology based or other interventions for loneliness a resource and tool is required which can effectively design such interventions for loneliness. This study aims to build the loneliness map as a tool and pool of resources which will have innovative sources of data such as social media, surveys, news sources, and other academic analyses to have data on loneliness. A pool of resources for studying loneliness such as policies, policy statements, and interventions etc. will also be provided as resources to better guide interventions for loneliness.

There are several studies which carry out comparative analysis among countries. Such comparisons point out the strengths and weaknesses of legal and executive functions and practice and provide an opportunity to identify gaps in a particular country’s legal and executive framework. Such comparisons have been carried out between USA and India too in multiple fields, varying from economics to healthcare systems. A. Khurrana et al. [12] carried out a comparative study of pharmacovigilance through the legal parameters and frameworks among EU, India, and the US. The study identified good pharmacovigilance practices and identified potential gaps in the frameworks. Similarly, J. M. Engel provided a comparison of various cardiac arrhythmias in India and USA through data collected from a mobile cardiac telemetry system [13]. The study identified various demographics which are at risk of conditions. V. Sathyanarayanan et al. [14] did a comparative study of access to novel drugs for Lymphoma and Leukemia between USA and India. The study took different parameters into consideration before concluding that the treatment of the conditions has the same impact on quality of life and patient outcomes in both countries.

C. Are et al. [15] pointed out disparities in cancer care between USA and India and suggested avenues to take the lead in addressing the disparities. Other areas of healthcare than direct medical care is also studied via comparative analysis between India and USA. C. Wright [16] compared pharmacy education in both countries and suggested ways through which both countries can efficiently and better utilize their trained pharmacists to improve patient care. The cultural impact of new technologies is also studied as for instance, N. Homburg carried out a cross-country comparison of attitudes toward humanoid robots in Germany, USA, and India [17]. New technology such as robots are affecting healthcare and such comparative cultural studies toward new technology can also give significant insights into healthcare.

This paper builds on the literature review of both technological interventions for loneliness and the comparative study of healthcare. The novel insights into the dynamic nature of loneliness gained from this paper will also contribute to the comparative analysis of healthcare across different countries.

## Data processing and analysis

This study is exempted from seeking ethical as this study analyzes publicly available online content. The study does not use identity of persons involved in generating the data but gives an aggregate and an overall picture based on opinions expressed publicly. We use social intelligence analysis (SIA) to find the correlation of loneliness with mental health problems and other correlated topics. The SIA is a broad theme which incorporates multiple social media sources such as Facebook, Reddit and Quora etc. SIA is important to gain insight into user’s data and in our case understand the dynamics of loneliness. While SIA can be used for a variety of purposes such as mining content to create stories or to find out trends, we have used SIA for sentiment analysis of collected data on loneliness.

SIA is important because of the vast availability of social media data. In today’s interconnected world, social media platforms and the web have become integral parts of our daily lives. As more people turn to these digital platforms for social interactions, it has become crucial to analyze the impact of social media on individual well-being, particularly in relation to loneliness. There are multiple reasons for such an SIA for loneliness: Firstly, social media provides a rich source of data that can offer insights into individuals’ social interactions, emotional states, and online behaviors. By analyzing this data, researchers can gain a deeper understanding of the factors contributing to loneliness and develop targeted interventions. This facilitates the identification of patterns, trends, and potential risk factors associated with loneliness. Furthermore, such analysis can provide valuable information for policymakers, healthcare professionals, and social media companies to develop evidence-based strategies and interventions to address loneliness in the digital age.

**Fig. 1:**
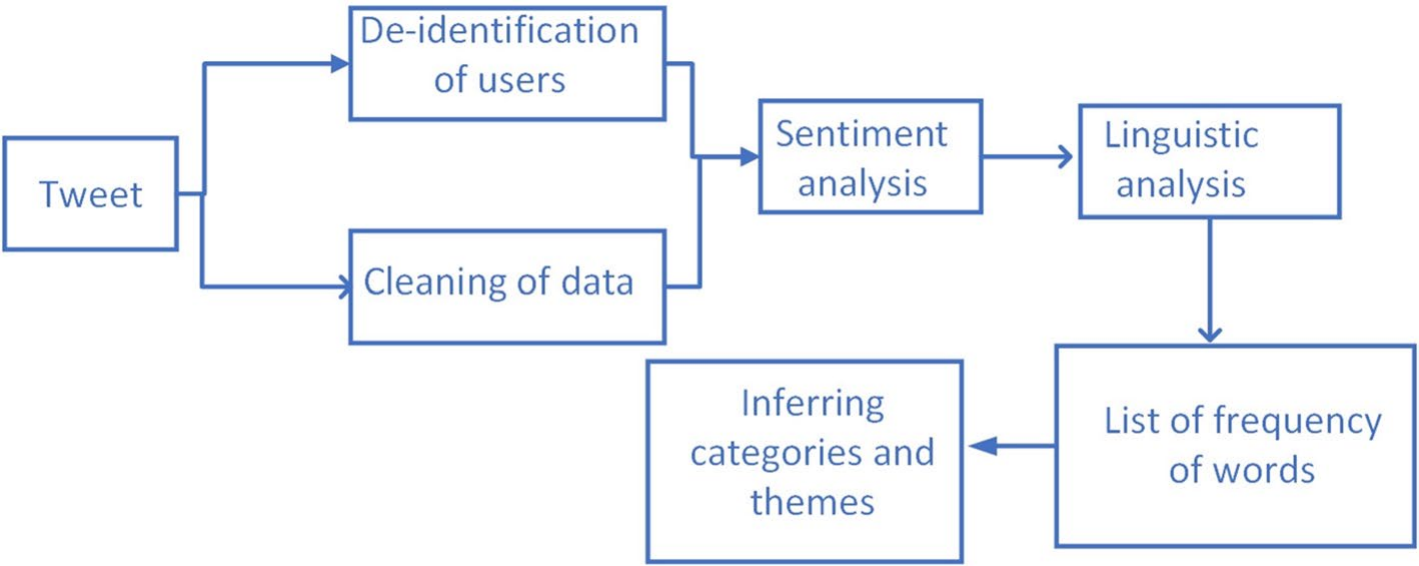
Pipeline for processing Twitter data

We used respective analysis of publicly available data of users posting about loneliness. Figure 1 presents our pipeline of analysis of data collected from Twitter. Twitter is a social media platform which is used for connectivity and opinion sharing and allows users to post via short messages consisting of 280 characters. Twitter gives access to the users’ data through its publicly available Twitter API for developers. The relevant tweets about loneliness were gathered and stored in a database. We created categories which are a combination of relevant words and phrases. These words and phrases convey meanings which can belong to the same broader division of sociopolitical or emotional-personal categories. After sentiment analysis, the tweets with negative sentiments were further analyzed by counting the occurrence of each word. The highly occurring words were then reported if they were conveying a meaning.

We used the words “lonely”, “loneliness”, “alone”, “isolated”, and “isolation” to give a list of tweets containing these keywords. We did not want to exhaustively search for one specific country because we wanted the data collected to be proof of concept. We can focus exhaustively on the cities or countries and collect more data about them based on the data collected in this step. In theoretical literature, the words “loneliness” and “lonely” are used to describe the feeling under consideration in this paper. Authors in [18] collected Twitter data based on the keywords “lonely” and “alone”. We went further and also included the synonyms and related words with loneliness for collecting our Twitter data.

If we were reporting all the tweets that contained feelings of loneliness, we would not have required a further step. In our case, the problem becomes determining the association between themes (which may represent loneliness) with the keywords depicting loneliness. For instance, we had to find what is the relationship between “hurt”, “sick”, “tired”, “sleep” etc. with the expression of loneliness. This task is usually carried out by the association of lexicon categories with tweets including the words “lonely” or “alone”. To carry out this task we use sentiment analysis. Sentiment analysis was carried out after cleaning the data such as removing redundant characters, numbers, special characters, users’ profile IDs and information such as “retweet”.

We stored the tweets with negative sentiments separately to carry out further analysis. We focused on negative sentiment tweets as the collected tweets also contain metaphorical use of lonely or loneliness which do not pertain to our use of loneliness. Such mention of loneliness is represented by the positive and neutral sentiment tweets. Further, we carried manual analysis of the negative sentiment tweets to find out relevant topics and themes. Stop words were removed before this step. We found out the number of occurrences of each word and phrase in the negative sentiment tweets. The manual analysis of the list of occurrences resulted in devising the larger socio-economic or emotional-personal categories. These categories will provide insight into the relationship between various emotional-personal and socio-political themes and the expression of loneliness and insomnia on social media.

This method of searching for relevant categories of sociopolitical and personal-emotional content and topics was used because it has more flexibility. Usually the n-gram method gives association of words that co-occur which cannot be perceived to have happened by chance. However, that method would not give the occurrence of individual topics and words, thus the impact of a topic would not be known.

### Sentiment analysis

For finding tweets with negative sentiment, we used VADER based on Python’s natural language processing toolkit (NLTK). VADER is suited for microblog content, such as that of Twitter. VADER combines lexicon, i.e., dictionary based analysis, and rule-based approach to characterize the sentiment. Other lexicon-based sentiment analyzers such as linguistic inquiry and word count (LIWC) [19] are only polarity based. VADER on the other hand also gives valence of the sentiment on the range from 1–9. Because of the sentiment score we can also know through VADER the extent to which the sentiment is negative or positive.

This valence is based on generalizable rules that represent grammatical and syntactical conventions that humans use in contexts meant for emphasizing sentiment intensity. For our purposes, another important feature of VADER is the inclusion of sentiment bearing lexical non-verbal items such as emoticons and verbal items such as slang, acronyms, initialisms which are prevalent in social media contexts. The combination of valence polarity through both lexicon and rule-based approach are valuable for fine-grained sentiment analysis. The shortcomings of lexicon-based approach include coverage, general sentiment intensity and acquiring a new set of human lexical features.

### Data collection and analysis

Tweets were collected containing the keywords mentioned in the last subsection. The collected data on the keywords contained 4.3 million tweets. The data was collected in October 2022 through the developer API of Twitter. The collected data was merged based on location, user ID and Tweet ID to identify tweets belonging to USA and India. The result was 1.6 million unique data frames for USA and the same number of data frames were selected for India. Five cities from each country with the highest number of tweets and data frames were identified and analyzed. It has to be noted that the cities analyzed had data frames of more than 10,000 each.

The number of Tweets to analyze was not determined on a specific rule. In literature, a varying number of tweets and Reddit posts are used for analysis. For example, A. Zubaiga [20] carried out an analysis of the collected Twitter dataset to show the diversity of subjects and the dataset sizes. There were datasets ranging from over a hundred thousand to more than 10 million. The authors believed that the aim of this paper is to reflect in indicative terms the difference in the use of social media as per the expression of loneliness, therefore the numbers selected here were reasonable. We collected data for a week in October to give us a glimpse of activity about the expression of loneliness. This is not exhaustive dataset of loneliness, but it extends beyond a spot capture and contains internal diversity when it comes to the period of day and number of days.

## Results

**Fig. 2:**
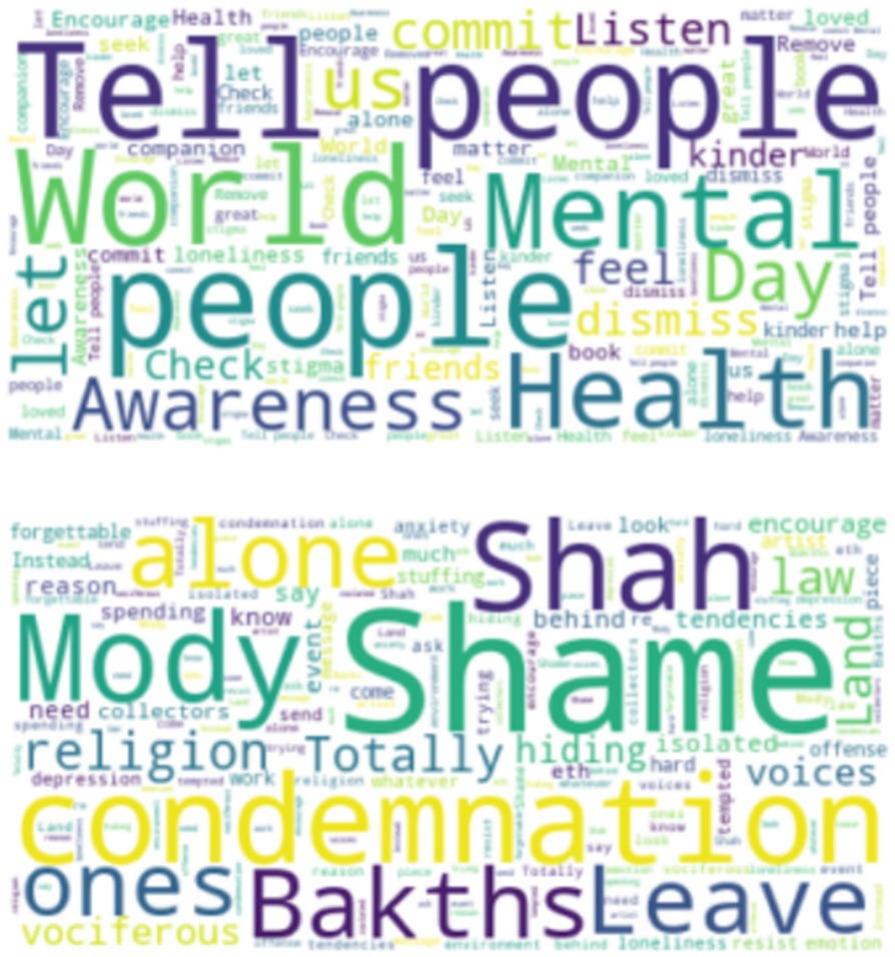
Words more likely to be posted by Twitter users in India (Top) when the sentiment of the tweet is positive, (Bottom) when the sentiment of the tweet is negative

**Fig. 3:**
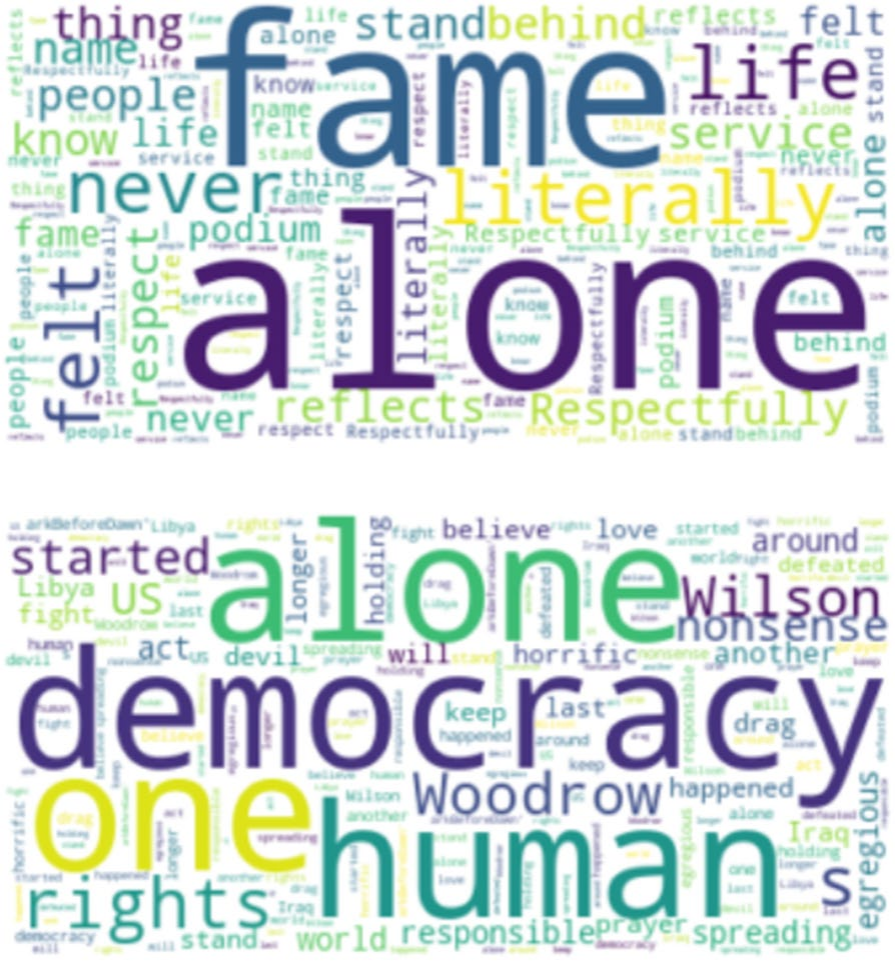
Words more likely to be posted by Twitter users in the USA (Top) when the sentiment of the tweet is positive, (Bottom) when the sentiment of the tweet is negative

Figures 2 and 3 represent the most frequent words associated with the mention of loneliness for India and USA respectively. These figures are called word clouds. Although the tweets were obtained using the loneliness keywords as mentioned in the previous section, the words in these figures can be seen as highly occurring topics with loneliness. Both in Figs. 2 and 3 the top word cloud is that of topics and themes associated with the positive mention of loneliness, while the bottom word clouds represent topics associated with the negative mention of loneliness. These can be important to get a general overview of the cause or correlation and association of loneliness.

**Fig. 4:**
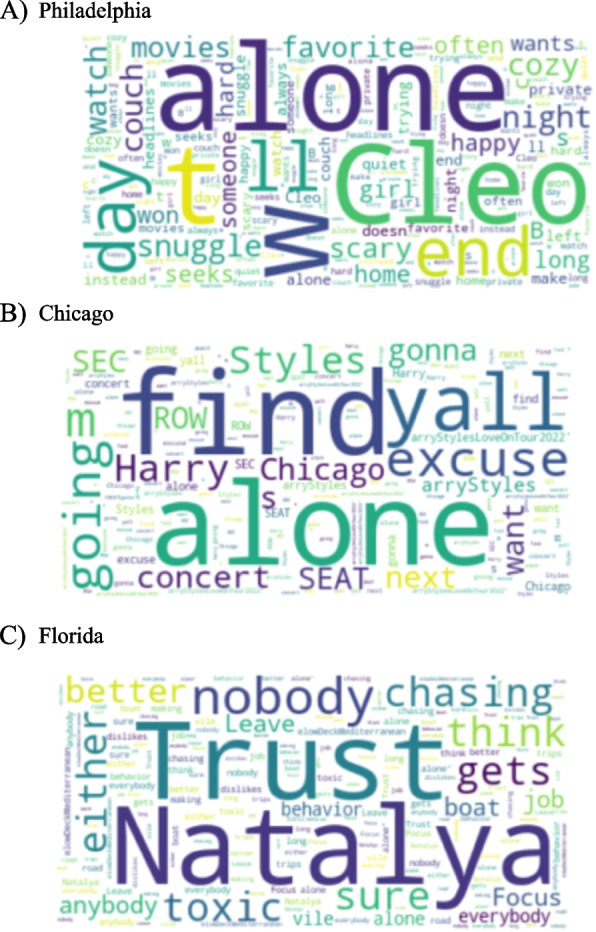
Words more likely to be posted by Twitter users when sentiment of the tweet is negative across sample cities in the USA

**Fig. 5:**
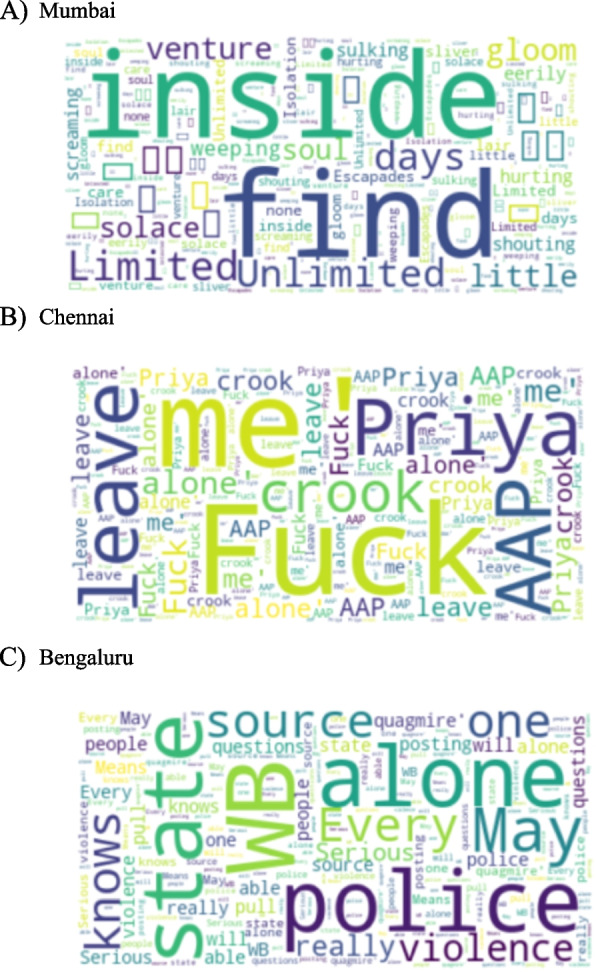
Words more likely to be posted by Twitter users when sentiment of the tweet is negative across sample cities in India

Figures 4 and 5 give word clouds of different cities across USA and India of words and topics which are highly occurring with negative mention of loneliness. These word clouds are made from tweets with negative sentiments with the mention of keywords of loneliness. The point of these figures of different cities is to prove that even within the same country the associated themes with loneliness can vary. The themes can vary from socio-political to personal-emotional across the geographical divide. For example, in the USA cities, the themes are personal-emotional with the occurrence of words such as ‘toxic’, ‘snuggle’, and ‘trust’, while for India it is mostly political with mention of words like ‘politics’, ‘violence’ and mention of political parties and political figures.

Table 1 gives sentiment analysis for both countries as explained above. The results show that there is a significant number of tweets that are neutral. In the case of India, it is more than half of the total Tweets. It means that these are the tweets that do not add meaning to our analysis of loneliness. The aim of the loneliness map and this paper is to find the correlation between loneliness and mental health issues and other topics which can vary from personal expression to socio-economic factors. Before going into a detailed analysis of the tweets on loneliness, it was important to find out if the tweets are metaphorical or non-sequitur. The neutrality can also represent the mention of loneliness in descriptive terms. Therefore, only the Tweets with negative sentiments associated with them must be analyzed to derive topics and themes which occur highly with loneliness.

**Table 1:** Sentiment analysis of Tweets containing the keywords of loneliness

Country	Sentiment analysis of Tweets
India	Positive: 8.3%
	Negative: 33.8%
	Neutral: 57.9%
USA	Positive: 23.7%
	Negative: 48.3%
	Neutral: 28.0%

Table 2 presents the highly correlated topics with negative mentions of loneliness in the USA. The tweets with negative sentiment were first tokenized and stemmed to get a concise list of words and topics associated with loneliness. The list was then analyzed and meaningful words representing topics of interest such as emotional, social and health etc. identifiers were found out. Words like ‘oh’, ‘yeah’, and ‘ur’ were ignored in composing the list. From Table 2 it can be seen for the overall US dataset intimate relationships followed by interpersonal relationships are the highest correlated topics, thus, issues associated with loneliness. “Death” as a topic representing matters of health also occurs the most in mention related to loneliness.

**Table 2:** Highly correlated topics with mentions of loneliness in the USA

**Highly correlated topics with negative mentions of loneliness. Topics are divided into a broader theme area**		
**Thematic area**	Topic	No. of mentions
**Intimate Relationships**	Cheat	13,540
	Family	7379
	Woman	8153
	Relationship	33,061
	Marriage	3781
**Interpersonal Relationships**	Want	31,333
	Need	37,233
	Feel	15,168
	Hurt	5344
	Forgave	4465
**Health**	Covid	3714
	Die	35,478
	Life	16,358
	Patient	1435
**Socio-economic factors**	Colorism (Black/White)	19,199
	Money	8368
	Poor	1880
	Racism	1383
**Emotional expression/insecurities**	Sad	8942
	Hate	11,035
	Fat	8701
	Anger	3525
	Grieve	2115
**Drug/Alcohol**	Rehabilitation	8580
	Smoke	3604
	Drunk	1289
**Insomnia**	Night	8415
	Awake	1402
	Sleep	9009

Similarly, Table 3 was obtained for highly correlated topics with negative mentions of loneliness in India. There are some similarities in information about loneliness between India and USA. However, the composition of the thematic categories also varies for both the countries. There was no political category in the USA mentioned but we had to accommodate the highly frequent occurrence of political words and themes into a new category. Likewise, we were not able to find a significant mention of themes associated with drug or alcohol intake with negative mention of loneliness. There was also high variance for other topic categories such as in “Insomnia” where the topic “Night” is mention with much higher frequency for India than for the USA. Interestingly the Intimate Relationships category for India does not have a high mention as is the case in the USA.

**Table 3:** Highly correlated topics with mentions of loneliness in India

**Highly correlated topics with negative mentions of loneliness. Topics are divided into a broader theme area**		
**Thematic area**	Topic	No. of mentions
**Intimate Relationships**	Family	7797
	Children	64
	Son	64
	Woman	64
	Relationship	113
**Interpersonal Relationships**	Want	1803
	Need	1719
	Feel	4163
	Hurt	113
	Despair	09
**Health**	Covid	11,313
	Die	8721
	Life	4091
**Socio-economic factors**	Injustice	4068
	Money	1344
	Poor	3391
	Culture	4452
	Right	3277
**Emotional expression/insecurities**	Sad	113
	F*ck	18,480
	Hate	45
	Anger	113
**Political**	War	4098
	Protect	4068
	Visa	8721
	Modi	8996
	Citizen	2085
	Custody	8721
	Muslim	904
	Hindu	226
	Protest	4068
**Insomnia**	Night	27,184
	Day	9435
	Sleep	1344
	Awake	1269

We have combined topics which we think belong to a category. The individual topics or words which appear on the Figures but not in the tables may be because they couldn’t fit into a broader category and, thus not included. Our concern here was to know the impact of wider categories rather than individual words/topics. The words that appear in the Figures are individual occurrences while Tables 2 and 3 present the topics which are grouped into categories. Moreover, Tables 2 and 3 present the combined analysis for each country of the tweets with negative sentiment. In Table 3, which is for the list of associations of negative sentiment tweets of India, we can still find some words which are mentioned in Fig. 2 (bottom), i.e., in the figure depicting the word cloud of negative sentiment tweets for India. For example, words like “Need”, “Mody” (variation of Modi), and “religion” (which in the Table are broken down into “Hindu” and “Muslim”) are present both in the Figure and the Table. However, for the Tables, we took words which were adding meaning to the socioeconomic and personal-emotional categories rather than the occurrence of individual and standalone words.

Tables 4 and 5 represent the top correlated topics across different cities of USA and India respectively. It was found that the sample, however limited, contained variation as per themes and topics associated with the negative consequences of loneliness. While these topics and their association with loneliness are not definitive, i.e., it may change with the availability of more data per city, it provides proof of concept for the idea of mapping loneliness, nonetheless. The data analyzed here provides a peek at data collected over a limited period, but it proves that the expression and dynamics of loneliness can change with geography which in turn can be dependent upon particularly urban infrastructure, healthcare system, socio-economic issues, and culture of the region. These two tables prove that even with limited data the dynamic nature of loneliness and its varying characteristics from one place to another or from one socio-economic and political context to another can be seen. As pointed out, these characteristics and associations are not definitive, but they do provide directions for research into these themes in particular geographical regions for further research.

**Table 4:** Top correlated topics with negative mention of loneliness across cities analyzed in USA

**City**	**Top 3 correlated topics**
**Atlanta**	1. Emotional expression (Leave)
	2. Emotional expression (Need)
	3. Life
**Chicago**	1. Leave
	2. Emotional expression (Want/Need)
	3. F*ck
**Florida**	1. Relationship
	2. Emotional expression (Hate)
	3. Reject
**Houston**	1. Relationship (Left/Cheat)
	2. Family
	3. Emotional expression (Sad)
**Philadelphia**	1. F*ck
	2. Trust
	3. Relationship (Man)

**Table 5:** Top correlated topics with negative mention of loneliness across cities analyzed in India

**City**	**Top 3 correlated topics**
**Bengaluru**	1. Emotional expression (Understand)
	2. Emotional expression (Need)
	3. Life
**Mumbai**	1. Environment (Rain, thunder, showers, cloud)
	2. Politics (Protest, Protect)
	3. Socioeconomic (Culture)
**Lucknow**	1. Socioeconomic (Wage)
	2. Politics (War, Fight, citizen)
	3. Relationship
**Chennai**	1. Politics (Visa, Modi, Religion)
	2. Health
	3. Year
**Chenglaruputtu**	1. Culture
	2. Work
	3. Relationship (Family)

While Figs. 2, 3, 4 and 5 give the word clouds and represent the frequency of occurrence of the top words, Tables 2, 3, 4 and 5 are composed after manually looking the lists of stemmed words and finding out meaningful words and patterns. In this way Tables 2, 3, 4 and 5 are based on subjective reading of the lists obtained for each country and city, i.e., they are not computed through programming code from the datasets. However, to find out meaningful connections of themes and topics with loneliness it is important to look up the frequency list manually. There are other words and phrases which occur more than the mentioned topics in these tables, but they are part of the grammar and do not convey any meaningful information.

The association of different topics such as politics, emotional and personal categories with loneliness are presented in Tables 2 and 3. Even in Tables 4 and 5, the association of different themes across different cities in USA and India can be seen. We have multiple topics associated with loneliness such as Health, Politics, Intimate Relationships and Socioeconomic factors etc. However, the data we have gathered is not suitable to predict the association of loneliness with certain events such as violent acts or shift in political opinion etc. Knowing it will require longitudinal data on loneliness on the association of different topics with loneliness. This will require collecting data throughout months and years. This is out of the scope of this work. The aim of this paper is to investigate whether there is any difference geographically in the expression of loneliness. The aim was not to present an exhaustive account of the expression of loneliness. To present an exhaustive analysis of loneliness containing all the events and topics associated with loneliness would require data collection on a much larger scale.

The problem of data collection leads us to another of the reviewer’s questions that why we chose October 2022 to collect the data. We started data collection in October 2022. We aimed to collect data for a week, to have some kind of temporal diversity in our data. We agree that this temporal diversity is not as much as required by the dynamic nature of loneliness, but this data gave us a glimpse into multiple factors such as insomnia. We could not extend this data because, in February 2023, it was announced by Twitter that there would be charges to data collection through Twitter API. The charges to collect the data are so high that without very strong organizational support, we could not continue collecting more data. For instance, collecting 1 million tweets would cost 5,000 USD. Because of this we could not extend our data collection and had to analyze the already collected data to infer the geographical differences in expression of loneliness through Twitter.

## Discussions and limitations

The methodology developed in this paper shows the association of loneliness with language which is associated with mental health issues such as anger and depression. The tweets analyzed prove that psychosocial linguistic features can be found in the self-expression of loneliness which can identify the dynamics of loneliness [21, 22]. Further, we present the topics and themes associated with loneliness can vary along both the thematic area and the geographic region. Tweets containing keywords associated with loneliness also represent a self-focused discourse which affirms previous literature on loneliness [23, 24].

Tables 2 and 3 also point toward other results which have been established in literature on loneliness. These include conformity with literature on the association of loneliness with substance abuse, emotional dysregulation, and trouble with relationships [25]. Moreover, for the case of India and its cities the political association of loneliness is a concept which is also studied in literature. In [26] a study of political participation and its relationship with loneliness was carried out. The data from Twitter for India also shows this political connection with loneliness when it comes to India. The results in this paper show that loneliness is a varied experience and can have multiple correlations varying from one place to the other. A loneliness map developed by social intelligence analysis through machine learning and social media data analysis can thus be a powerful tool for policymakers.

The analysis of the tweets gave us attributes to know the associations of socioeconomic and personal-emotional factors with loneliness. These attributes include emotion, sentiment, emojis, and topics. This analysis demonstrated that such attributes could help gather evidence and analyze interactions on the topic of loneliness and other such related topics. The first attribute was emotion, which can serve as a guide in understanding people’s reactions. The second most common attribute was relationships. Other thematic areas such as health, work, self-focused topics and insomnia related topics indicate the intimate nature of loneliness.

Finding correlations with mental health and other issues in public health such as loneliness through social media analysis is interesting because such analysis can give insight into first-hand experiences of people. The question for this paper was is there a significant difference in the geographical expression of loneliness? The answer to this question is not a binary one. When it comes to larger patterns of association of topics with loneliness, there are similar patterns. However, there are differences as to the emphasis or nature of themes associated with loneliness.

The similarities and differences between the expression of themes associated with loneliness for India and USA can be because of a variety of reasons. Authors in [27] found that the causes and associations of loneliness vary across geographical regions. The finding was that to a significant extent loneliness can be determined by induvial characteristics, but the regions do tend to contribute to the nature of causes of loneliness as per their findings. Individuals living in socioeconomically disadvantaged areas may have less avenues of leisure or higher quality of life while in some places like in urban areas, the loneliness may be associated with lack of greenery and nature. As per the literature, the causes and associations of loneliness can vary. This paper also provides a data based affirmation of this finding.

The similarities between USA and India in expression of loneliness are qualitatively the same for most themes and categories. The difference mostly is that of quantity of the impact of each category. As discussed, this can be the result of specific geographical and regional features. It is an important aspect of loneliness that although the level of association with a theme can vary there are some themes which can be present everywhere, or for the matter of this study, both in India and USA. Some other thematic categories and expression topics such as political ones and drug use can be regional in nature. Therefore, this paper gives a prototype of a larger model of associations and combined theory of loneliness using data.

This paper has given some insights into the dynamics of loneliness such as:

1. Despite the limitations of the dataset, the varying nature of loneliness across geographic dimensions can be seen. The results of city-wise analysis also provide the nature of varying themes and topics associated with loneliness.
2. As can be seen through the addition of different category of “Political” for analysis of loneliness in India, there is no uniform criteria of social themes and topics associated with loneliness. At best we can have a geographical or otherwise demographic association of socio-economic themes and concerns with loneliness.
3. Social intelligence analysis, in this case analysis of Twitter data on loneliness, can reflect the actual social conditions. The example of this is in India where the emphasis is on political terms of isolation. This can be in reference to the polarized politics of the country and the debate ensuing around different political policies and actions.

There are some limitations of this study. The first limitation is that the dataset size is very small as compared to the actual data being generated by both countries on the keywords of loneliness. Tweets can run into millions even for a city on the keyword of loneliness. But the purpose of this study is not to carry out a rigorous analysis but to point the direction for further investigation into loneliness. This study also aims to prove that loneliness is felt differently, which is proved by the data collected, analyzed and the results obtained. The second limitation of this study is that the data from Twitter can be combined with other data sources on loneliness such as data from other social media sites such as Facebook and reddit etc. Similarly, clinically validated data on loneliness or data from surveys can be used to calibrate the results obtained in this study. For this data to be scientifically sound and be the basis for solid policy interventions these other data sources considerations need to be addressed. The limitation of this study on another front is the automatic classification through of Tweets into negative and positive through sentiment analysis. While this has been the basis of the paper to carry out automated analysis, the result of this automated sentiment analysis needs to be validated through looking at a certain number of Tweets which have been identified negative. Through this way, we will be able to know the confidence of analysis and quantify the error.

## Conclusion

This paper develops the proof of concept for loneliness map project. In this proof of concept, we carried comparative analysis of USA and India through data collected from Twitter to see the correlation of loneliness with negative sentiment and other correlated topics. The comparative analysis of both the countries is carried out for a variety of public health topics of interest. This paper adds to the comparative literature on public health and develops a global public health map on loneliness. Loneliness map is not only meant to see the prevalence of loneliness in different countries, regions, and cities around the world, but it will also be instrumental in understanding the impact of different socio-cultural, political, economic, and geographical dynamics on loneliness and mental health. The loneliness map can guide intervention for policy makers in healthcare like the health map in (www.healthmap.org). The interventions can also be guided by data provided by the loneliness map.

In this paper sentiment analysis of tweets containing keywords associated with loneliness was carried out for US and India. The results showed variance in the sentiment associated with loneliness in different cities as well as the top correlated topics with the mention of loneliness. These results are only indicative and will need further exhaustive study. To point out for the sake of clarity, the number of tweets containing the keywords associated with loneliness can run up to millions for a particular city during a year. But the objective of this paper is not to study exhaustively each city but to determine from the data collected the sentiment associated with loneliness to prove that the dynamics of loneliness are not the same and can vary from country to country and even can vary in the same country for different geographical locations. The comparative analysis in this paper drives the point home that loneliness can be varied and would need different strategies to counter the negative feelings associated with loneliness.

## Data Availability

The datasets generated and/or analyzed during the current study are not publicly available due to the online resource for which the data is utilized is under construction. The data and materials of the paper will be publicly available through the map in future but are available from the corresponding author on reasonable request.
